# 359. The Clinical Utility of the GenMark Dx ePlex® Fungal Blood Culture Identification Panel

**DOI:** 10.1093/ofid/ofac492.437

**Published:** 2022-12-15

**Authors:** Stefania Carmona, Jeremy Meeder, Derek Moates, Todd P McCarty, Rachael A Lee, Sixto M Leal

**Affiliations:** University of Alabama at Birmingham, Birmingham, Alabama; University of Alabama at Birmingham, Birmingham, Alabama; University of Alabama at Birmingham, Birmingham, Alabama; University of Alabama at Birmingham, Birmingham, Alabama; University of Alabama at Birmingham, Birmingham, Alabama; University of Alabama at Birmingham, Birmingham, Alabama

## Abstract

**Background:**

The GenMark ePlex® Fungal Blood Culture Identification (BCID) Panel utilizes electrowetting technology to detect 15 most common causes of fungemia. Rapid identification of fungal species and innate resistance patterns enable improved antifungal stewardship.

**Methods:**

In this prospective study, aliquots of the initial diagnostic blood culture bottle with fungal organisms detected on Gram Stain (n=61) received standard of care (SOC) fungal identification in two study periods. MALDI-TOF MS was utilized in both phases. BCID-FP results were not reported to treating clinicians during the pre-implementation phase. After 35 isolates, BCID-FP results became part of the SOC for all bloodstream infections (implementation phase) with results available to providers. Chart reviews were performed to assess risk factors for candidemia and evaluate the potential then actual impact of the BCID-FP on the time to organism identification, treatment, and patient outcomes.

**Results:**

A total of 61 patients were included in the final analysis, 35 in the pre-implementation phase and 26 in the post-implementation phase (Table 1). *C. albicans* was most common, followed by *C. glabrata* and *C. parapsilosis*. The cohort includes two cases of *Cryptococcus* as well as two rare yeasts unable to be identified by BCID-FP and requiring the state lab identification (Table 2). Overall outcomes and differences between groups are seen in Table 3. The BCID-FP identified species 1.4 days faster compared to SOC methods across all patients, 1.12 days in the pre-implementation phase vs. 1.81 days in the post-implementation phase. In 32 patients (52%), the BCID-FP allowed for an earlier change in antifungal therapy for species with known low risk of fluconazole resistance.

**Figure:**
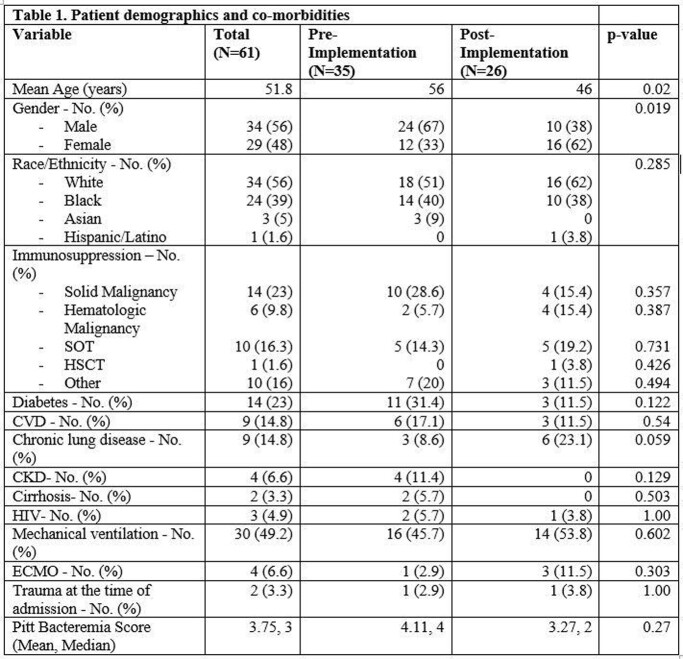

**Figure:**
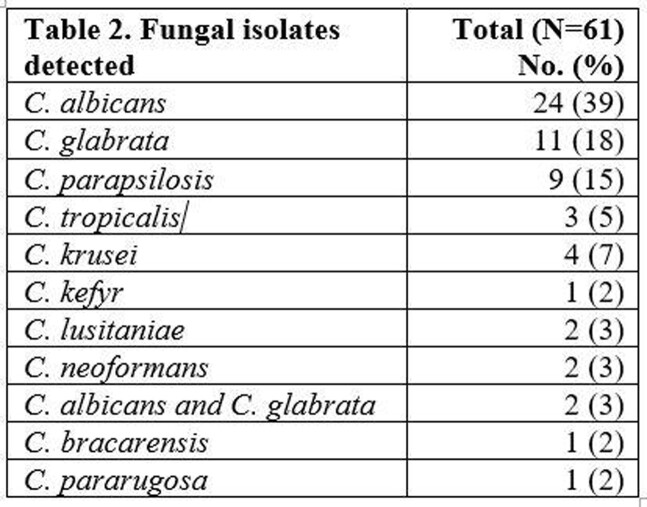

**Figure:**
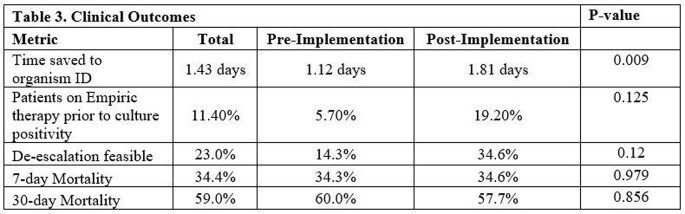

**Conclusion:**

The BCID-FP enabled earlier fungal identification compared to SOC identification. Earlier identification allows for earlier antifungal stewardship as well as better empiric therapy for non-*Candida­* fungal pathogens. Empiric therapy rates were low with high mortality rates, indicative of an ongoing need for improving the care of patients with fungemia.

**Disclosures:**

**Todd P. McCarty, MD**, GenMark Dx: Grant/Research Support|GenMark Dx: Honoraria **Sixto M. Leal, Jr., MD, PhD**, GenMark Dx: Grant/Research Support|GenMark Dx: Honoraria.

